# A novel homozygous mutation in the solute carrier family 12 member 3 gene in a Chinese family with Gitelman syndrome

**DOI:** 10.1590/1414-431X20165261

**Published:** 2016-10-24

**Authors:** Y. Zhang, F. Zhang, D. Chen, Q. Lü, L. Tang, C. Yang, M. Lei, N. Tong

**Affiliations:** 1 Division of Endocrinology and Metabolism, West China Hospital, Sichuan University, Chengdu, Sichuan, China; 2 School of Clinical Medicine, Chengdu University of Traditional Chinese Medicine, Chengdu, Sichuan, China; 3 School of Clinical Medicine, West China Hospital, Sichuan University, Chengdu, Sichuan, China

**Keywords:** Gitelman syndrome, *SLC12A3* gene, Homozygous mutant, Pedigree

## Abstract

Loss of function of mutated solute carrier family 12 member 3 (*SLC12A3*) gene is the most frequent etiology for Gitelman syndrome (GS), which is mainly manifested by hypokalemia, hypomagnesemia and hypocalciuria. We report the genetic characteristics of one suspicious Chinese GS pedigree by gene sequencing. Complete sequencing analysis of the *SLC12A3* gene revealed that both the proband and his elder sister had a novel homozygous *SLC12A3* mutation: c.2099T>C and p.Leu700Pro. Moreover, the *SLC12A3* genes of his mother and daughter encoded the same mutated heterozygote. It was noted that in this pedigree, only the proband complained about recurrent episodes of bilateral lower limb weakness over 8 years, while his elder sister, mother and daughter did not present symptoms. The inconsistent clinical features of this pedigree implied that besides diverse phenotypes possibly originated from the same genotype, gender difference may also dominate the variant GS phenotypes. Further genetic and proteomic research are needed to investigate the precise mechanisms of GS, including the study of specific ethnicities.

## Introduction

Gitelman syndrome (GS), an autosomal recessive inherited disease (OMIM 263800) first reported by Gitelman in 1966, manifests mainly as hypokalemia, hypomagnesemia and hypocalciuria (1–5). Some patients also suffer from abdominal pain, weakness, muscular hypotonia, periodic paralysis, and myoclonus, as well as other manifestations of hypokalemia (6). Because the clinical phenotype of GS shows great heterogeneity, GS patients suffer from a diverse range of clinical symptoms (7).

GS is caused by mutations in the solute carrier family 12 member 3 gene (*SLC12A3*), which is 55,000 nucleotides long, is composed of 26 exons, and is localized in chromosome 16q13 (3). Mutations in *SLC12A3* impair the function of the sodium-chloride co-transporter (NCCT) of the distal convoluted tubule (DCT) (4,8).

The NCCT is an integral membrane protein of 1,021 amino acids with 12 transmembrane domains, which is responsible for the reabsorption of 5-10% filtered sodium and chloride in the DCT. Loss of NCCT function is thought to explain the clinical manifestations of GS.

Apart from GS, other conditions such as hyperthyroidism and primary hyperaldosteronism can also present with hypokalemia and episodic muscular weakness. Hyperthyroidism causes muscular weakness that is associated with electrolyte disturbances and signs of thyrotoxicosis, whereas the muscular weakness of hyperaldosteronism is associated with hypertension, hypokalemia, and elevated plasma aldosterone levels (9,10 [Bibr B11]). Because the clinical symptoms and laboratory findings of GS are not typical in many cases, genetic analysis is considered the most precise method for GS diagnosis, although currently there appears to be no correlation between genotype and phenotype within GS patients (7,8,).

Here we report a case of a patient who presented with generalized weakness, persistent hypokalemia, hypocalciuria, hypomagnesemia, and hypochloremic alkalosis, all of which are consistent with the typical manifestations of GS. Upon further investigation, he was found to have a novel mutation in *SLC12A3*: c.2099T>C and p.Leu700Pro, which was also found in other members in this pedigree. To the best of our knowledge, this mutation has not been previously reported or collected in any gene database.

## Case Report and Methods

### Case Report

A 24-year-old male was admitted to the clinic due to recurrent episodes of bilateral lower limb weakness over 8 years, which had been aggravated during the past month. Eight years previously, he began experiencing bilateral lower limb weakness and gait instability, without pain or other symptoms, which were relieved by the oral or intravenous daily administration of 4–10 g of potassium supplementation. He experienced similar episodes approximately every 2 years.

One month before presenting to our clinic, the patient had rebound weakness upon the discontinuation of potassium. He was admitted to the hospital for further diagnosis. On admission, he was alert and oriented, with no sign of anemia. His vital signs were a temperature of 36.2°C, a pulse rate of 93 beats per min, 20 breaths per min, and a blood pressure of 127/87 mmHg. His waist circumference was 95 cm, and his body mass index was 26.12 kg/m^2^. His past medical history was positive for hepatitis B virus infection. No similar findings were present in his mother, elder sister, or his daughter. Family members had no history of consanguineous marriage, use of laxatives or diuretics, or alcohol abuse. This study conformed to human research guidelines stated in the Declaration of Helsinki, and all subjects gave their written informed consent.

### Methods

*Laboratory tests*. All the laboratory parameters were determined by Laboratory Medicine Department of West China Hospital, Sichuan University. Serum and urine electrolytes were tested by Cobas8000 automatic biochemical analyzer (Roche, Switzerland). Arterial blood gas was analyzed by GEM3000 blood gas analyzer (Instrument Laboratory, American). Thyroid hormones were measured by electrochemiluminescence immunoassay. Plasma renin activity and aldosterone levels were detected with radioimmunoassay.

*Gene analysis*. The proband's father died several years before and we were unable to reach his wife, so we could not collect samples of their DNA. Other family members provided peripheral blood samples, which were used to extract DNA with the QIAamp DNA BloodMini Kit (Qiagen, USA). Gene amplification was performed by polymerase chain reaction (PCR) of 3 ng/mL DNA samples with the Ion AmpliSeq™ Library Kit (Life Technology, USA). PCR was conducted using *SLC12A3* primers (listed in Supplementary Table S1) on a GeneAmp 9700 PCR system (ABI) under the following conditions (8,12,[Bibr B13] 14): 5-min predenaturation and 30-s denaturation at 95°C, annealing at 57-64°C, and 90-s chain extension at 72°C; the above steps were recycled 30 times. All sample densities were of 3 ng/mL. Gene sequencing was performed and analyzed by ABI Personal Genome Machine (Life Technology). In addition, multiple ligation-dependent probe amplification (MLPA) was used to identify small base insertions or deletions. Gene amplification and sequence analysis were conducted at Division of Endocrinology and Metabolism, West China Hospital and Kingmed Center for Clinical Laboratory, Sichuan. The analysis of amino acid conservation was conducted according to the UniProt database. Three different *in silico* prediction tools, including PolyPhen 2, LRT and MutationTaster, were used to predict whether this mutation was pathogenic.

## Results

### Clinical manifestations and laboratory results

Dynamic blood pressure measurements found that the proband's mean arterial blood pressure was within normal range. Serial complete blood counts, liver function tests, and renal function tests did not display any abnormal findings (data not shown). Endocrine investigations determined that his thyroid hormones and aldosterone levels were normal. Serum electrolyte analysis demonstrated persistent hypokalemia and hypomagnesemia, while his urine electrolyte analysis demonstrated persistent hypocalciuria. Blood gas analysis demonstrated persistent metabolic alkalosis. Table 1 presents a summary of these results.

**Table:**
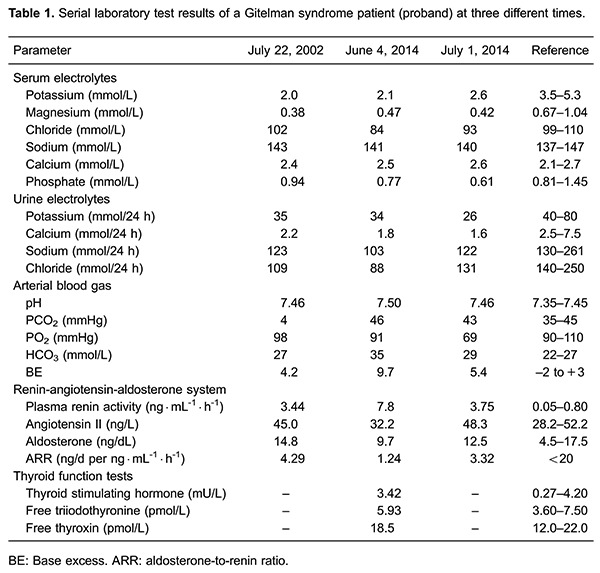

### Genetic diagnosis

**Figure 1:**
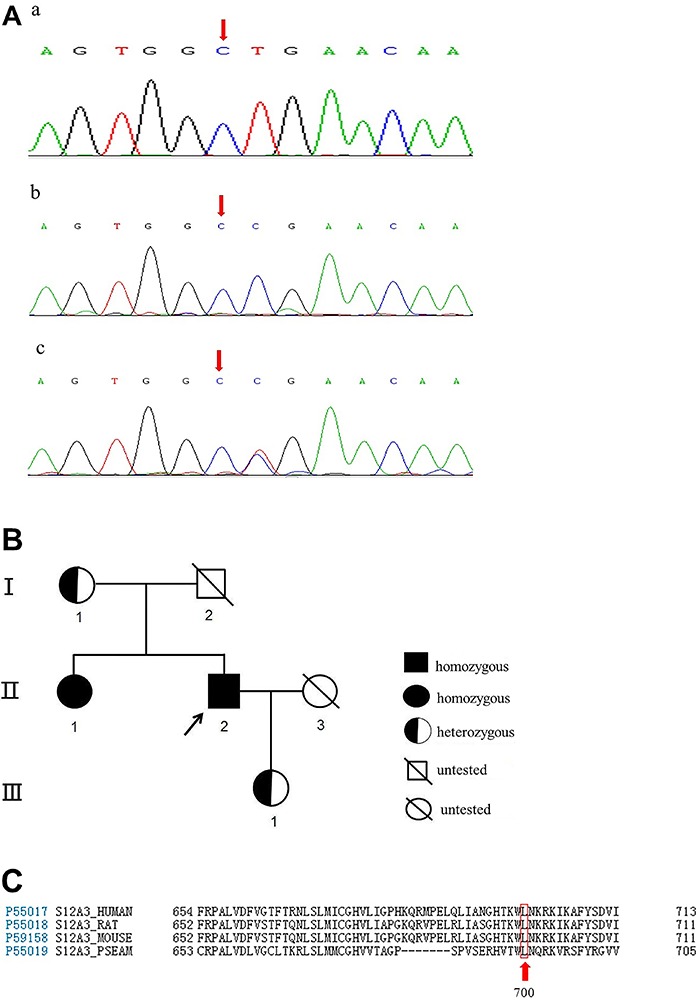
*A*, mRNA sequences diagram of mutant *SLC12A3* gene in the studied pedigree: *a*, mRNA sequences of wild type; *b*, mRNA sequences of the patient's mother and daughter, who have heterozygous mutations (in exon 17, c.2099T > C, CTG CCG, leading to Leu700Pro); *c*, mRNA sequences of the patient and his sister, who have homozygous mutations. *B*, family pedigree chart. *C*, analysis of amino acid conservation across various species (human, rat, mouse and *Pleuronectes americanus*), indicating that the mutation (amino acid residue 700 encoding leucine) was located in the highly conserved amino acid sites.

Because the clinical features of this patient were indicative of GS, we carried out genetic analysis, which identified a novel mutation in *SLC12A3*. Specifically, the mutation was at the 2099^th^ nucleotide position and involved the substitution of a thymidine residue by a cytosine residue (2099T>C) resulting in the substitution of leucine by proline at codon 700 of the NCCT (Figure 1A,c). MLPA showed that no insertions or deletions were present in any of the 26 exons of *SLC12A3*. Subsequent genetic evaluation of *SLC12A3* in the patient's mother, sister, and daughter revealed the identical mutation at the same locus (Figure 1A,b), and the same homozygous variant was also detected in the patient's sister (Figure 1A,c). The pedigree chart is presented in Figure 1B. The analysis of amino acid conservation across various species (human, rat, mouse and *Pleuronectes americanus*) indicated that this mutation was located in the highly conserved amino acid sites (Figure 1C). Furthermore, the *in silico* analysis results revealed that this novel genetic mutation in *SLC12A3* was deleterious (Table 2).

**Table:**
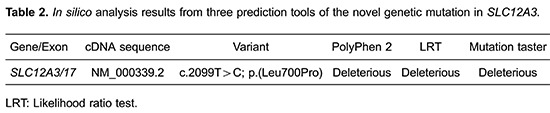

## Discussion

We describe a Chinese pedigree carrying a homozygous *SLC12A3* mutation resulting in the substitution of leucine by proline at codon 700 of the NCCT. The proband was a young male who had been suffering from bilateral lower limb weakness for 8 years. His clinical manifestations and laboratory tests suggested a diagnosis of GS, which was confirmed by genetic analysis. Interestingly, his elder sister carried the same homozygous mutation but was asymptomatic; moreover, his mother and daughter also carried a mutated allele, and were asymptomatic.

Though GS is an autosomal recessive salt-losing renal tubulopathy, its heterozygote prevalence is not rare. Indeed, a Swedish and Italian population studies found that the heterozygote prevalence of GS is approximately 1% in European populations (8). Tago et al. (15) reported an overall frequency of heterozygous NCCT mutations of 3.21% using TaqMan analysis of 1852 Japanese subjects, and a GS morbidity of 0.103% in Japan. Similarly, Lin et al. (16) found an overall incidence of positive heterogeneous mutations of approximately 3% after detecting 12 NCCT mutations in 100 unrelated healthy Chinese subjects. The authors also identified nine mutations that were potentially common in Chinese GS patients, including T60M and H90Y. These findings show that GS is a highly heterogeneous disease, and that it has a frequency of 1:40,000 in the Caucasian population (17). Moreover, besides mutations in *SLC12A3*, loss of function of the chloride channel CLC-Kb gene has also been shown to be causative of GS (18).

To date, 428 mutations throughout *SLC12A3* have been collected in the Human Gene Mutation Database, including 269 missense/nonsense, 58 splicing, 47 small deletions, 22 small insertions, five small indels, 22 gross deletions, four gross insertions, and one complex rearrangement. Previous studies have identified at least five potential mechanisms for the inactivity of the NCCT transporter: class 1 mutants exhibiting decreased protein stability, class 2 with impaired protein processing, class 3 showing the disrupted insertion of other functional proteins into the plasma membrane, class 4 with a reduced sodium uptake rate, and class 5 with accelerated protein removal or degradation (19,20). Thus, *SLC12A3* mutations cause structural changes and/or dysfunction of NCCT, resulting in disturbances of the tubular reabsorption of sodium and chloride ions, and finally, the development of GS. Since the *SLC12A3* mutation (2099T>C) of this pedigree gave rise to the substitution of leucine by proline at codon 700 of the NCCT, we inferred that the location is near the C-terminal domain of the 12-transmembrane NCCT segments. However, the exact mechanism for this *SLC12A3* mutation needs further genetic and proteomic research to elaborate the possible genotype-phenotype correlations of GS.

In the current pedigree, only the proband suffered from the bilateral lower limb weakness of hypokalemia, while other family members were asymptomatic. This could be explained by the fact that GS is more severe in patients with two mutant alleles than those with only one mutated allele (21). Indeed, patients with homozygous mutations are reported to have more severe symptoms than those with heterozygous mutations, as well as an increased risk of developing chronic kidney damage and diabetes mellitus (22). This is also in line with the characteristics of autosomal recessive inheritance.

However, this does not explain the disparate phenotypes observed between the proband and his elder sister. As an autosomal recessive disease, the GS incidence should not be influenced by gender. However, several previous studies have shown that female GS patients/carriers are much less severely affected than male GS subjects (6,7,23). As we have previously reported on a pedigree carrying a novel *SLC12A3* gene homozygous mutation of GS in three patients with the homozygous mutation, the male proband also had more severe clinical symptoms and laboratory findings, and a poorer prognosis than the other two female relatives (14). These evidences suggest that gender may play a crucial role in phenotypic diversity. Cruz et al. (6) also observed that GS symptoms could be influenced by premenopausal or postmenopausal status in women, in whom estrogen levels varied greatly. Therefore, although the underlying mechanism has not been clarified, it might be explained by a positive effect of estrogen on NCCT regulation (24). This prompts us to speculate that estrogen-related therapy could be used as a candidate approach to treat GS. However, further experimental and clinical data are needed to investigate this possibility.

In conclusion, we identified a novel *SLC12A3* mutation in a Chinese GS pedigree, leading to the substitution of leucine by proline at codon 700 of the NCCT transporter. The proband and his elder sister had a homozygous mutation, while his mother and daughter carried one mutated allele. Because only the proband suffered from bilateral lower limb weakness, we inferred that the same genotype manifests as diverse phenotypes. Additionally, a gender effect may influence the GS symptom severity. To achieve a more thorough understanding of GS, further genetic and proteomic research is needed to investigate the precise mechanisms of GS, including research of specific ethnicities.

## Supplementary material

Click here to view [pdf].
